# Positioning an ECG electrode to the dorsal side can record higher amplitude of CMAPs during cryoballoon ablation

**DOI:** 10.1002/joa3.12314

**Published:** 2020-02-13

**Authors:** Kazuya Mizukami, Tsuneaki Homma, Hiroyuki Natsui, Mizuki Kato, Keisuke Otsu, Takashi Takenaka, Minoru Sato

**Affiliations:** ^1^ Department of Cardiovascular Medicine National Hospital Organization Hokkaido Medical Center Sapporo Japan

**Keywords:** atrial fibrillation, compound motor action potential, cryoballoon, phrenic nerve injury, pulmonary vein isolation

## Abstract

**Purpose:**

Phrenic nerve injury (PNI) is one of the important complications during cryoballoon (CB) ablation. Recording diaphragmatic compound motor action potentials (CMAPs) during CB ablation can predict PNI. CMAP monitoring may be inaccurate when CMAP amplitudes are low. We examined the effect of positioning an electrocardiography (ECG) electrode at the dorsal side.

**Methods:**

We retrospectively analyzed the cases of 197 consecutive patients who underwent CB ablation for pulmonary vein isolation (PVI) (April 2016 to December 2018) at our institution. CMAP amplitudes were monitored using two recording methods just before cryoapplication. (a) Conventional method: right‐arm ECG electrode positioned 5 cm above the xiphoid on the ventral side; left‐arm ECG electrode positioned along the costal margin. (b) Our original method: right‐arm electrode positioned 5 cm above the xiphoid on the dorsal side; left‐arm electrode positioned along the costal margin.

**Results:**

The CMAP amplitude during right phrenic nerve pacing was significantly higher at the dorsal side than the ventral side (0.80 ± 0.31 mV vs 0.66 ± 0.29 mV, *P* < .01). Similarly, the CMAP amplitude during left phrenic nerve pacing was significantly higher at the dorsal side than the ventral side (0.92 ± 0.39 mV, 0.73 ± 0.37 mV, *P* < .01). PNI occurred in six patients (3.0%); three patients experienced transient PNI, another three patients experienced persistent PNI, and none developed permanent PNI.

**Conclusions:**

CMAP amplitudes were significantly high at the dorsal side compared to the ventral side. Monitoring phrenic nerve function using an ECG electrode at the dorsal side is a simple and easy procedure.

## Graphical abstract

The amplitude of diaphragmatic compound motor action potentials was significantly high at the dorsal side of the xiphoid compared to the conventional ventral side. Monitoring phrenic nerve function using an electrocardiography (ECG) electrode at the dorsal side is a simple and easy procedure.

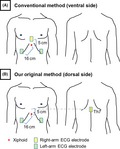

## 1 BACKGROUND

Atrial fibrillation (AF) is one of the most common cardiac arrhythmias, and its prevalence increases with age.^1^ It is thus expected that the incidence of AF will increase dramatically in today's aging populations. Pulmonary vein isolation (PVI) is now one of the most effective treatments for AF.^2^, ^3^ A common method used for PVI is the use of a radiofrequency current. However, in 2016, ablation with a cryoballoon (CB; Arctic Front Advance^®^, Medtronic) was shown to be non‐inferior to radiofrequency ablation for the treatment of patients with paroxysmal AF, and the procedure duration and left atrial dwell time were short in the CB ablation patients compared to those who underwent radiofrequency ablation.^4^ The number of CB ablations for AF has thus been increasing.

Phrenic nerve injury (PNI) is a rare but important complication of CB ablation.^5^ Ghosh et al reported that rapid balloon deflation results in more rapid tissue rewarming, leading to prevention of persistent PNI.^6^ Therefore, continuous phrenic nerve stimulation during CB ablation is important to reduce the risk of phrenic nerve palsy. One method of the active monitoring of the phrenic nerve is monitoring of the diaphragmatic compound motor action potentials (CMAPs). During CB ablation, recording the CMAPs on a modified lead I can be used to predict PNI.^7^, ^8^ Some studies reported that using CMAP surveillance during CB ablation decreased the incidence of PNI.^9^, ^10^, ^11^, ^12^

However, PNI is still cause for concern in CB ablation, and CMAP monitoring may be inaccurate if the CMAP amplitude is low. We conducted the present study to examine the effect of the positioning of an electrocardiography (ECG) electrode at the dorsal side, that is, opposite to the site 5 cm above the xiphoid.

## 2 METHODS

We retrospectively analyzed the cases of 197 consecutive patients who underwent CB ablation for PVI in the period from April 2016 to December 2018 at our institution. Written informed consent was obtained from all patients. Transesophageal echocardiography was performed before PVI in each patient to determine and exclude the presence of any atrial thrombi. Computed tomography of the left atrium (LA) was performed to examine the PV anatomy. Patients received general anesthesia with propofol combined with dexmedetomidine hydrochloride. The study was approved by the Ethics Committee of National Hospital Organization Hokkaido Medical Center.

### 2.1 Ablation procedure

A single transseptal puncture was performed under fluoroscopic and intracardiac echocardiographic guidance (AcuNav, Biosense Webster). Thereafter, heparin was administered intravenously to maintain an activated clotting time over 300 seconds. The second‐generation 28 mm CB catheter was introduced into the LA through a steerable sheath (FlexCath Advance, Medtronic). A mapping catheter (Achieve, Medtronic) was advanced within the CB to the PV. The CB was inflated and advanced to the ostium of each PV. Cryothermal energy was applied for 180 seconds with the CB. The order of CB application was as follows; left superior PV (LSPV), left inferior PV (LIPV), right inferior PV (RIPV), and right superior PV (RSPV). If necessary, PV isolation was completed with additional CB applications or an irrigated radiofrequency catheter.

### 2.2 CMAP recording

**Figure 1:**
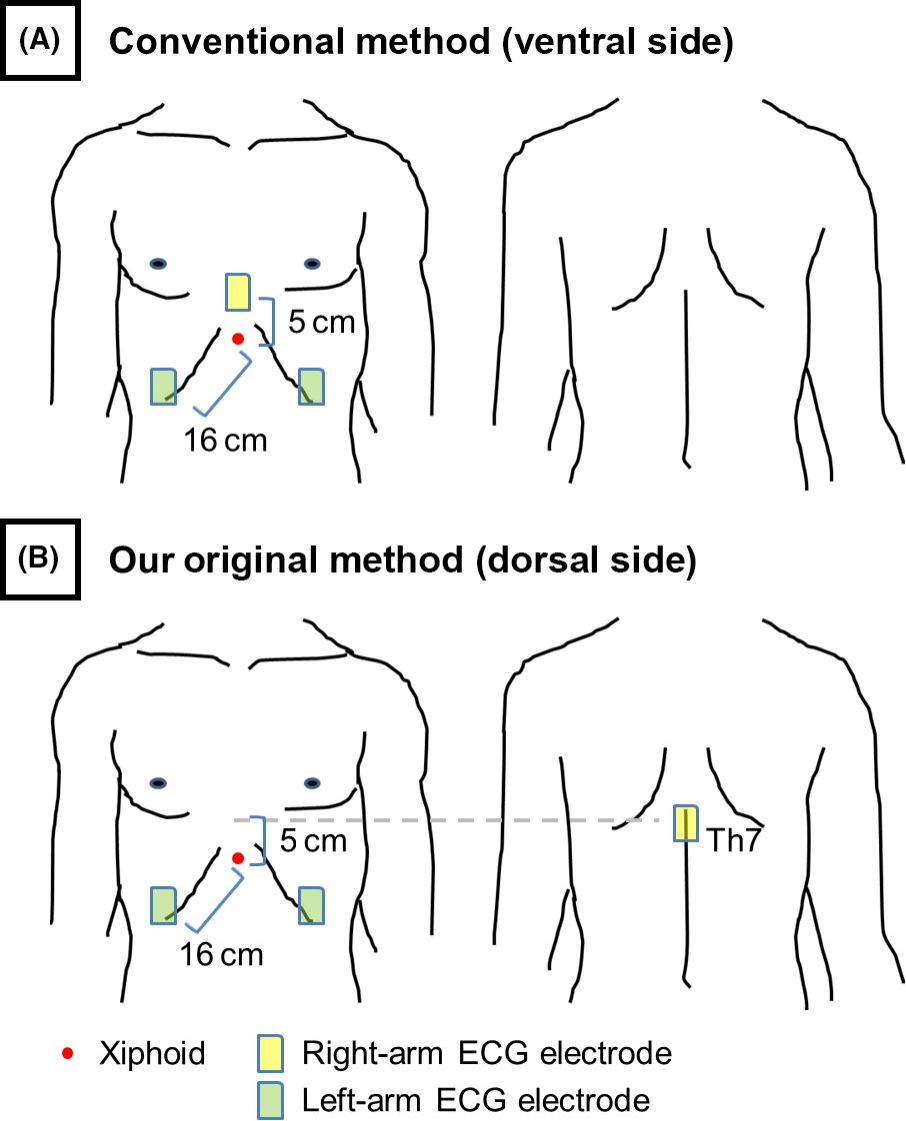
Position of the electrocardiography (ECG) electrodes. The compound motor action potential (CMAP) amplitude was monitored with two recording patterns just before cryoapplication. One pattern was the conventional method (ventral side): a right‐arm ECG electrode was positioned 5 cm above the xiphoid; a left‐arm ECG electrode was positioned 16 cm from the xiphoid along the right and left costal margins (A). The second recording pattern was done with our original method (dorsal side): a right‐arm ECG electrode was positioned on the thoracic vertebrae of the same level of the conventional method (ie, Th7); a left‐arm ECG electrode was positioned at the same position as that in the conventional method (B)

The CMAP amplitude was measured with two recording patterns before CB application. One pattern was the conventional method (ventral side): a right‐arm ECG electrode was positioned 5 cm above the xiphoid; a left‐arm ECG electrode was positioned 16 cm from the xiphoid along the right and left costal margins (Figure 1A).^9^ The second recording pattern was done with our original method (dorsal side): a right‐arm ECG electrode was positioned on the thoracic vertebrae of the same level of the conventional method (ie, Th7); a left‐arm ECG electrode was positioned at the same positions as that in the conventional method (Figure 1B).

Before CB application of the right or left PVs, the circular catheter (Optima, St. Jude Medical) was positioned to the superior vena cava or left subclavian vein and each phrenic nerve was paced (7.0 V/2 msec at 40/min). CMAP signals were amplified using a band‐pass filter setting between 0.5 and 300 Hz and recorded on a NavX (St. Jude Medical) recording system. CMAP amplitudes were measured simultaneously with two methods in NavX recording system before CB application. Each mean value of five phrenic nerve stimulations was compared. CMAP amplitude with the higher method was monitored continuously during CB application because it was impossible that the two CMAP amplitudes were monitored simultaneously and continuously in NavX recording system. If the 30% reduction in the amplitude of CMAP was reached, CB application was immediately discontinued.

To clarify the factors affecting CMAP amplitude before CB application, we divided the patients into three groups by CMAP amplitude, (a) Low; CMAP amplitude at the dorsal side was lower than 0.05 mV at the ventral side, (b) Same; the difference between CMAP amplitude at the dorsal side and at the ventral side was less than 0.05 mV, (c) High; CMAP amplitude at the dorsal side was higher than 0.05 mV at the ventral side. We compared the characteristics of three groups.

PNI was diagnosed by manual palpation of the hemidiaphragm during phrenic pacing, by fluoroscopy surveillance of diaphragmatic contractions, or an elevated hemidiaphragm on postprocedural radiography. Normalization of the chest radiography was considered a sign of complete clinical recovery. PNIs were classified by duration; PNI resolved immediately or on discharge was transient, PNI resolved within 12 months after hospital discharge was persistent, and PNI continued after 12 months was permanent.

### 2.3 Statistical analysis

Continuous data are expressed as the mean ± SD. The significance of differences in the average CMAP amplitude was determined using a paired *t* test. For multiple‐group comparisons, one‐way ANOVA followed by the Bonferroni's test was performed. A value of *P* < .05 was considered statistically significant. All statistical analyses were performed with EZR (Saitama Medical Center, Jichi Medical University).^10^

## 3 RESULTS

The characteristics of the 197 patients are summarized in Table 1. A total of 794 PVs were identified, and 728 of the 794 PVs were isolated by CB alone. Touch‐up ablation was needed in the remaining 66 PVs. The total number of CB applications was 1220:268 for 197 RSPVs, 319 for 196 RIPVs, 333 for 188 LSPVs, 253 for 187 LIPVs, 17 for 17 right middle PVs, and 30 for 9 left common PVs, and the mean number of CB applications was 1.4 ± 0.6, 1.6 ± 0.8, 1.8 ± 1.0, 1.4 ± 0.7, 1.0, and 3.3 ± 1.3 for left superior, left inferior, right inferior, right superior, right middle, and left common PVs, respectively.

**Table 1:** Characteristics

N = 197	
Age (years)	65.0 ± 9.8
Male	148 (75.1%)
Height (cm)	164.8 ± 9.1
Weight (kg)	68.1 ± 14.3
Body mass index	24.9 ± 3.9
Paroxysmal AF	126 (64.0%)
Persistent AF	52 (26.4%)
Longstanding AF	19 (9.6%)
Ejection fraction (%)	63.4 ± 10.2
Left atrial diameter (mm)	37.2 ± 6.2
Diabetes mellitus	29 (14.7%)
Hypertension	115 (58.4%)
Prior stroke or transient ischemic attack	14 (7.1%)
Heart failure	23 (11.7%)
Coronary artery disease	24 (12.2%)
CHADS2 score	1.1 ± 1.0
CHA2DS2‐VASc score	2.1 ± 1.5

Note

Data are given as mean ± SD or n (%).

Abbreviation: AF, atrial fibrillation.

**Figure 2:**
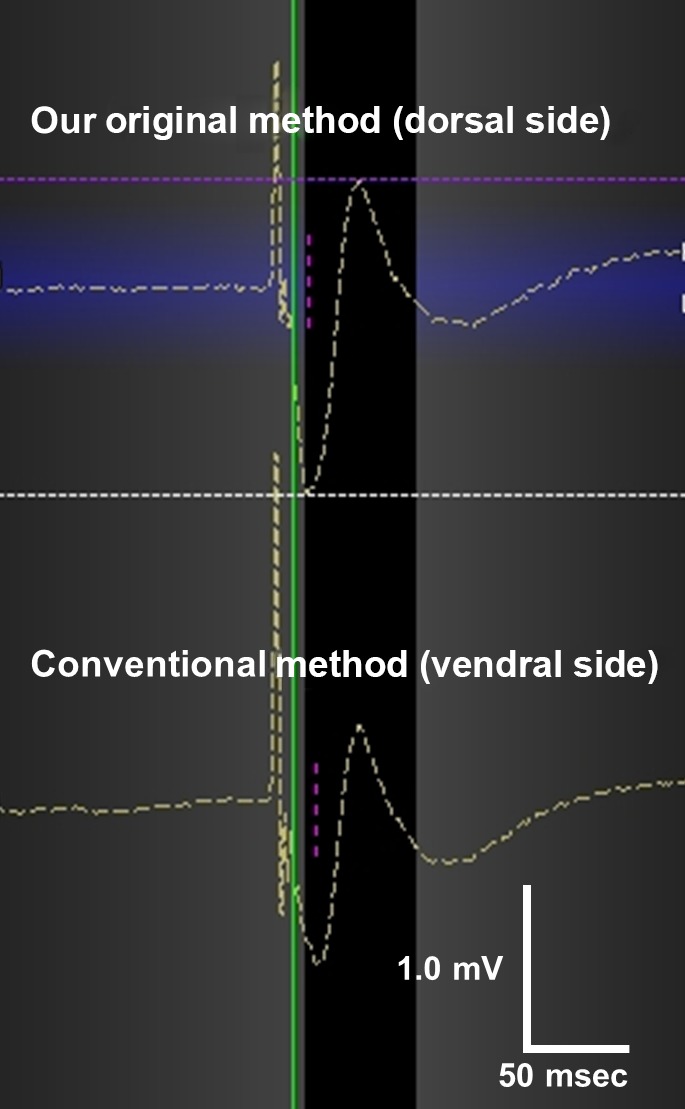
Compound motor action potential (CMAP) amplitude. CMAP amplitudes are measured simultaneously with the dorsal method (upper) and the ventral method (below) in NavX recording system before cryoballoon application

**Figure 3:**
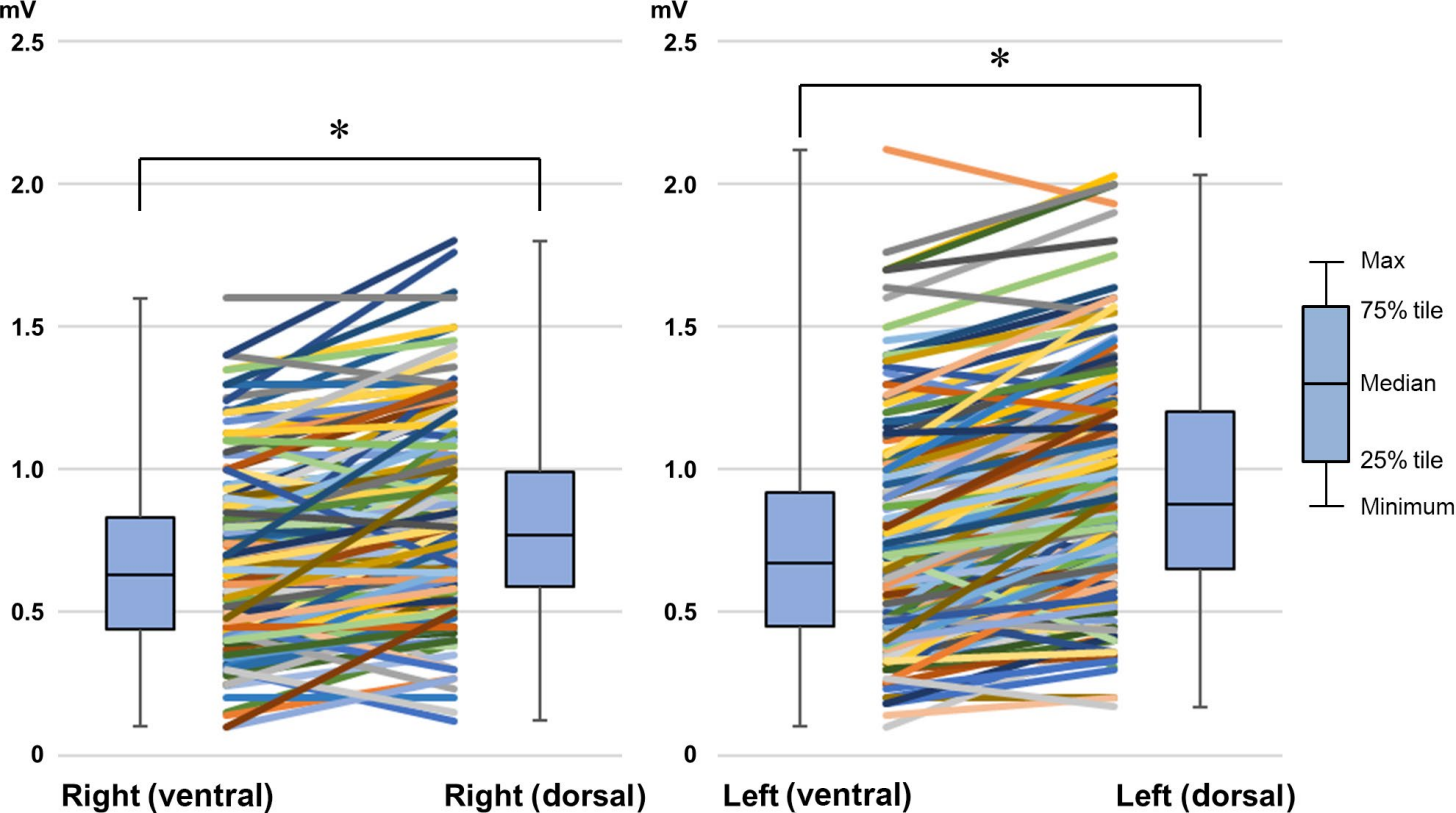
The average compound motor action potential (CMAP) amplitude during phrenic nerve pacing. Summary data for the CMAP amplitude during right and left phrenic nerve pacing. *P < .05

The dorsal and ventral CMAP amplitudes were measured simultaneously before CB application (Figure 2). The CMAP amplitude during right phrenic nerve pacing was significantly high at the dorsal side of the xiphoid compared to the conventional ventral side (0.80 ± 0.31 mV vs 0.66 ± 0.29 mV, *P* < .01). Similarly, the CMAP amplitude during left phrenic nerve pacing was significantly high at the dorsal side compared to the conventional ventral side (0.92 ± 0.39 mV, 0.73 ± 0.37 mV, *P* < .01; Figure 3). We monitored the CMAP amplitude with our original method in 169 of the 197 patients (85.8%) and in 179 of 197 patients (90.9%) during CB applications to right‐sided and left‐sided PVs, respectively. The characteristics of the patients in whom the CMAP amplitudes with the new method were low, almost same, or high compared to the conventional method are shown in Table 2. In majority, the CMAP amplitude was high at the dorsal side compared to the conventional ventral side. There were not significant differences between three groups.

**Table 2:** Characteristics of the patients in whom the CMAP amplitudes with the new method were low, almost same, or high compared to the conventional method

	Low	Same	High	*P* value
Right side				
N	22 (11.2%)	29 (14.7%)	146 (74.1%)	
Age (years)	69.2 ± 8.2	65.7 ± 10.7	64.2 ± 9.7	.07
Male	19 (86.3%)	23 (79.3%)	106 (72.6%)	.33
Height (cm)	164.9 ± 7.3	167.4 ± 8.2	164.3 ± 9.5	.26
Weight (kg)	65.9 ± 12.1	68.9 ± 14.9	68.2 ± 14.5	.73
Body mass index	24.1 ± 3.7	24.4 ± 3.9	25.1 ± 3.9	.43
Left side				
N	15 (7.6%)	9 (4.6%)	173 (87.8%)	
Age (years)	69.0 ± 8.1	67.4 ± 10.0	64.5 ± 9.8	.17
Male	14 (93.3%)	8 (88.9%)	126 (72.8%)	.13
Height (cm)	168.0 ± 9.3	167.3 ± 10.2	164.4 ± 9.0	.26
Weight (kg)	73.3 ± 17.8	70.8 ± 13.2	67.5 ± 13.9	.27
Body mass index	25.8 ± 4.7	25.2 ± 3.6	24.8 ± 3.8	.61

Note

Data are given as mean ± SD or n (%).

Abbreviation: CMAP, compound motor action potential.

The number of 30% drops in CMAP amplitude at each PV is shown in Table 3. In majority, the CMAP amplitudes returned to baseline value immediately after discontinuation of CB applications. PNI occurred in six patients (3.0%); three patients experienced transient PNI, another three patients experienced persistent PNI, and none developed permanent PNI. All persistent PNIs were resolved at 1 month after discharge. In all patients who developed PNI, the CMAP amplitudes dropped during RSPV cryoablation and did not return to baseline value after discontinuation of CB applications. Comparison of CMAP down (±) and PNI (±) during RSPV cryoablation is shown in Table 4. Freeze durations of CMAP down (+) and PNI (−) group and CMAP down (+) and PNI (+) group were shorter than CMAP down (−) and PNI (−) group. Pre‐CMAP amplitude of CMAP down (+) and PNI (+) group was lower than CMAP down (+) and PNI (−) group. Minimal amplitude and maximal CMAP drop of CMAP down (+) and PNI (+) group were lower than other two groups.

**Table 3:** The number of 30% drops in CMAP amplitude at each pulmonary vein

RSPV	16/197 (6.1%)
RIPV	10/196 (5.1%)
RMPV	0/17 (0%)
LSPV	0/188 (0%)
LIPV	4/187 (2.1%)
LCPV	0/9 (0%)

Note

Data are given as n (%).

Abbreviations: LCPV, left common pulmonary vein; LIPV, left inferior pulmonary vein; LSPV, left superior pulmonary vein; RIPV, right inferior pulmonary vein; RMPV, right middle pulmonary vein; RSPV, right superior pulmonary vein.

**Table 4:** Comparison of CMAP down (±) and PNI (±) during RSPV cryoablation

	CMAP down (−)	CMAP down (+)	CMAP down (+)	*P* value
	PNI (−)	PNI (−)	PNI (+)	
N	181 (91.9%)	10 (5.1%)	6 (3.0%)	
Characteristics				
Age (years)	65.1 ± 9.8	62.9 ± 9.5	65.7 ± 9.2	.78
Male	138 (76.2%)	6 (60.0%)	5 (83.3%)	.48
Height (cm)	165.2 ± 9.0	162.0 ± 9.4	158.8 ± 10.2	.14
Weight (kg)	68.3 ± 14.1	65.7 ± 16.5	63.7 ± 16.5	.63
Body mass index	24.9 ± 3.8	24.7 ± 3.8	24.9 ± 4.7	.98
RSPV cryoablation				
No. of freeze	1.3 ± 0.6	1.8 ± 0.6	1.3 ± 0.5	.06
Freeze duration (s)	178 ± 26	137 ± 45^a^	109 ± 25^a^	<.01
Minimal temperature (°C)	−52.7 ± 5.6	−51.2 ± 7.1	−47.8 ± 3.3	.10
Right‐sided CMAP during RSPV cryoablation				
Selected side (ventral/dorsal)	26/155	0/10	2/4	.20
Pre‐amplitude (mV)	0.86 ± 0.32	1.12 ± 0.52^a^	0.56 ± 0.26^b^	<.01
Minimal amplitude (mV)	0.80 ± 0.30	0.63 ± 0.29	0.17 ± 0.08^a^, ^b^	<.01
Maximal CMAP drop (%)	6.8 ± 7.1	43.6 ± 8.1^a^	67.3 ± 19.0^a^, ^b^	<.01

Note

Data are given as mean ± SD or n (%).

Abbreviations: CMAP, compound motor action potential; PNI, phrenic nerve injury; RSPV, right superior pulmonary vein.

a *P* < .05 vs CMAP down (−) and PNI (−) group.

b *P* < .05 vs CMAP down (+) and PNI (−) group.

## 4 DISCUSSION

Our analyses demonstrated that the CMAP amplitude at the new method (dorsal side) was significantly high compared to the conventional method (ventral side). This new method is a simple and easy procedure.

Although CB ablation has gained increasing attention as an effective ablation tool for PVI, PNI is an important complication during this procedure for PVI.^5^ The diaphragmatic CMAP is used to provide useful diagnostic information about phrenic nerve function in patients with neuromuscular disorders. The CMAP is the recorded summated muscle potential waveform produced by the innervated muscle fibers when the nerve is stimulated. Several studies have shown the clinical utility of monitoring CMAPs to anticipate PNI during CB ablation.^9^, ^11^, ^12^, ^13^, ^14^ In the present study, six patients who developed PNI showed a 30% drop in CMAP during RSPV cryoapplication, and the ablation was discontinued.

However, CMAP monitoring may be inaccurate in patients of low CMAP amplitude. If the CMAP amplitude is low, the safety margin of CMAP drop is very small and we do not easily detect these changes during CB ablation. It may cause the delayed discontinuation of cryoablation. In our study, pre‐CMAP amplitudes of the patients who developed PNI were lower than the patients who did not developed PNI in spite of dropped CMAP amplitudes during RSPV cryoablation. However, the number of patients who developed PNI was small in our study; further studies are necessary to determine that high CMAP amplitude can prevent PNI.

During CB ablation, the CMAP amplitude has usually been monitored with a right‐arm ECG electrode positioned 5 cm above the xiphoid and a left‐arm ECG electrode positioned 16 cm along the right costal margin. Because the CMAP is the recorded summated muscle potential waveform, high CMAP amplitudes can be monitored if there are many muscle fibers between the electrodes and the direction of the electrodes is the same direction as that of the muscle contraction. We thus hypothesized that the CMAP amplitude would be high at the dorsal side of the xiphoid compared to the conventional abdominal side. In fact, our analyses demonstrated that the CMAP at the dorsal side was higher in most of our patients' cases. As we used the CMAP amplitude at the dorsal side in over 85% of the patients, there was a low rate (3%) of PNI. In addition, this technique requires only one ECG electrode.

PNI is more common on the right side of the body because of the close anatomic relationship between the right PN and right PV. However, several reports have shown that left‐sided PNI could be provoked during a left‐sided CB ablation procedure.^15^, ^16^ An operator was therefore also needed to monitor the phrenic nerve function during CB ablation in order to prevent left‐sided PNI. Miyazaki et al showed that stable left‐sided diaphragmatic CMAP was obtained by pacing from the left subclavian vein during CB ablation.^14^ Recording the left‐sided CMAP could be useful to monitor left‐sided PN function. In the present study, stable left‐sided CMAP was obtained at the abdominal and dorsal sides, and four cases showed a >30% reduction in CMAP during the left‐sided CB ablation.

This study has several limitations. First, it was a retrospective study in a single center and might therefore incorporate important biases. Second, the number of patients was small. Third, we added only a single ECG electrode at the dorsal side. We did not test other configurations. Fourth, we did not directly compare the CMAP at the dorsal side to that at the ventral side during cryoapplication. Further studies are required to achieve better diagnostic accuracy using the CMAP to prevent PNIs. Fifth, we did not measure the threshold for phrenic nerve capture. If the threshold was close to 7V in many patients, the results might be affected by partial phrenic capture.

## 5 CONCLUSIONS

Our study revealed that the CMAP amplitude was significantly high at the dorsal side of the xiphoid compared to the conventional ventral side. Monitoring phrenic nerve function using an ECG electrode at the dorsal side is a simple and easy procedure. Further studies are necessary to determine the clinical impact of the new method of CMAP monitoring on reducing PNI during CB ablation for AF.

## CONFLICT OF INTERESTS

The authors declare no conflict of interests for this article.
